# Effectiveness of Oral Nutritional Supplements on Older People with Anorexia: A Systematic Review and Meta-Analysis of Randomized Controlled Trials

**DOI:** 10.3390/nu13030835

**Published:** 2021-03-03

**Authors:** Mengqi Li, Si Zhao, Shuang Wu, Xiufen Yang, Hui Feng

**Affiliations:** Xiangya School of Nursing, Central South University, Changsha 410013, China; mayinapril@sina.com (M.L.); zhaosi@csu.edu.cn (S.Z.); shuangwu@csu.edu.cn (S.W.); yangxiufen1995@csu.edu.cn (X.Y.)

**Keywords:** oral nutritional supplements, anorexia of aging, appetite, energy intake, body weight, systematic review

## Abstract

Background: Nutrition plays an important role in maintaining the overall health of older people. Inadequate intake may lead to impaired body function, higher morbidity, and mortality. Oral nutritional supplements (ONS) showed positive effect on the nutritional status of the elderly; however, systematic evidence is currently lacking on the effect of ONS on the elderly with anorexia. Aims: The current systematic review and meta-analysis included randomized controlled trial (RCT) articles to investigate the effectiveness of ONS on the main aspects of anorexia of aging (AA). Methods: By using the Preferred Reporting Items for Systematic Reviews and Meta-Analyses (PRISMA) method, researchers independently searched PubMed/MEDLINE, EMBASE, CINAHL, PsycINFO, the Cochrane Library, China National Knowledge Infrastructure (CNKI) and other gray literature resources for publications that met the inclusion criteria by October 2020. The Cochrane Risk of Bias Tools were used for quality assessment. The inverse-variance method was used for the fixed model (FM) while the DerSimonian–Laird method was used for the random model (RM). Respective 95% confidence intervals (95% CIs), mean difference (MD) or standardized mean difference (SMD) was used for indices in terms of effect size (ES). Results: 2497 records were found through the systematic search, while 17 RCTs (*n* = 1204) were included, with a mean age of 81.9 years (range: 74–87 years). Supplementation occurred in the morning, mid-day, and evening, while the times varied from one to three times a day. The results of meta-analysis showed that, generally, ONS had a positive effect on the overall appetite, MD = 0.18, 95% CI (0.03, 0.33), *p* = 0.02, and consumption, MD = 1.43, 95% CI (0.01, 2.86), *p* = 0.05; but not significant in terms of other aspects of appetite: hunger, *p* = 0.73; fullness, *p* = 0.60; desire to eat, *p* = 0.80; preoccupation, *p* = 0.15. Additionally, it showed an increase in the overall energy intake, SMD = 0.46, 95% CI (0.29, 0.63), *p* < 0.001, in protein intake, SMD = 0.59, 95% CI (0.16, 1.02), *p* = 0.007, and in fat intake, MD = 3.47, 95% CI (1.98, 4.97), *p* < 0.001, while no positive effect was found on carbohydrates intake, *p* = 0.06. Significance differences were also found in the body weight, SMD = 0.53, 95% CI (0.41, 0.65), *p* < 0.001, and body mass index (BMI), MD = 0.53, 95% CI (0.12, 0.95), *p* = 0.01. Moreover, subgroup analyses were conducted according to the nutrient density with no positive results showed except for the low-density ONS on overall energy intake. Conclusions: The results of the present study indicated that ONS had beneficial effects on overall appetite, energy intake, body weight and BMI.

## 1. Introduction

As an important public health problem with high prevalence around the world, malnutrition happens especially in those with neurological diseases, malignant tumors, and the older adults [1,2,3,4]. Malnutrition may adversely affects the clinical outcomes, and increases the economic cost of healthcare [5,6], while the standard treatment is still an issue to be discussed [7].

Anorexia of aging (AA) or senile anorexia (SA) is one of the major causes of malnutrition and has been identified as a geriatric syndrome [8,9,10,11]. It can be defined as the physiological loss of appetite that occurs with aging [12], leading to reduced intake of energy and nutrients [13,14], then causes weight loss [15,16,17,18]. Decreased appetite may lead to malnutrition, which is directly related to decreased body mass index and the development of pressure sores [10,19,20,21]; therefore, there might also a link between AA and the latter two aspects. Irreversible outcomes, such as a decrease in endurance, quality of life, physical function, immune function, cognitive function, and even sarcopenia and cachexia, may occur because of AA [18,21,22,23,24,25,26,27,28], but is often underreported [29].

Older people with AA tend to have lower energy intake and appetite perceptions physiologically in the fasted and postprandial states, although the mechanisms are not fully studied [30,31]. Studies pointed out that external cues such as changes of living environment and routine would all affect appetite [9]. It is believed that the development of AA relates to pathophysiological factors, for example, aging [15], changes in body compliance [11], increased anal sphincter muscle tension, and activity of cholecystokinin [8,32] would all lead to satiety and cause physiological anorexia [33,34].

Globally, the prevalence of AA is 20–30% [9,10], and is more common in older adults in hospitals and nursing home settings, with prevalence rates ranging from 23% to 62% [15]. The prevalence is about 10% higher in the elderly with neurological diseases such as dementia, inflammatory diseases such as chronic kidney disease [21,35]. The figures may continue to rise as the global population ages and lifespans increase.

Growing concerns about the adverse health impacts and high prevalence of AA has led to calls for effective treatment to avoid it at the early stage [29,36]. Among the treatments [5], oral nutritional supplements (ONS) are uniquely positioned.

Guidelines by the European Society for Parenteral and Enteral Nutrition (ESPEN) stated that enteral nutrition (EN) should be the first choice for older adults who are malnourished or at risk with normal or nearly normal gastrointestinal function [37,38]. As the primary choice for EN, the specialized nutritional supplement formulas of ONS can enhance the nutrient content of protein, carbohydrates, fats, minerals, and vitamins [37] with the characteristics of simplicity, convenience, and lower price, which meet the psychological desires of the elderly to consume orally. This energy-rich supplement form can compensate for the problem of homogeneous energy in natural meals, provide balanced nutrients to meet the demand for nutrients, and maintain or improve the nutritional status of patients. By taking between meals of 200–600 kcal per day [39], ONS can also be used as a normal diet or an addition for hospitalized or community-dwelling elderly [40], and the smaller the BMI (<18.5 kg/m^2^), the more it will benefit the elderly person [5,39,41,42,43].

However, significant disparities exist in the implementation of ONS in the elderly, greatly impairing the potential of ONS to achieve sufficient health outcomes in the nutritional status. Thus, it is important to identify and summarize the effectiveness of ONS on AA.

Ample literature illustrated the unique impacts of ONS in the treatment of older people [44], but mainly focus on the late malnutrition stage when the elderly can choose nothing but ONS as a diet [45], while there is limited research on ONS as a treatment for AA [36]. Elderly people with AA usually have good intestinal health, and compared to tube feeding, ONS is closer to the natural eating process and has better compliance [46].

The purpose of this systematic review and meta-analysis was to examine the effectiveness of ONS on AA, including the main features of appetite, intake, and body weight, meanwhile, body mass index, diarrhea, pressure sores, quality of life and the medical cost were also reviewed, with no limitation of the state of ONS (solid or liquid) or the settings in which the interventions conducted (community, hospital, or nursing home).

## 2. Materials and Methods

### 2.1. Study Protocol

The current review followed the Preferred Reporting Items for Systematic Reviews and Meta-Analyses (PRISMA) for the identification, screening, eligibility, and inclusion of studies [47], and has been registered on the International Prospective Register of Systematic Reviews (PROSPERO), registration number: CRD42019124227. The protocol is available online [48].

### 2.2. Data Sources and Search Strategy

Two experienced researchers (M.L. and S.Z.) developed search strategies in MEDLINE using the relevant free text and MeSH terms, then modified and developed search strategies for each database. The detailed search strategies are provided in the Supplementary Materials (see Table S1).

The same two researchers then independently searched and screened PubMed/MEDLINE, EMBASE, CINAHL, PsycINFO, the Cochrane Library, and China National Knowledge Infrastructure (CNKI), as well as the open gray databases for articles published up to October 2020. To minimize publication and language bias, there were no restrictions on publication year or language. Additional studies were identified by hand-searching of reference lists in previous reviews, websites (e.g., clinicaltrials.gov, ISRCTN Register, Current Controlled Trials (controlled-trials.com), Meta Register of Controlled Trials, and Trial Trove). Conference publications from BAPEN and ESPEN were searched for relevant abstracts. Disagreements during the extraction process were discussed and a consensus was reached in group meetings with the arbitrators (S.W. and X.Y.).

### 2.3. Intervention and Control Group Definitions

The intervention group was defined as participants who were on an oral supplementation diet (those with protein, fat, amino acids, and vitamins, etc.) for at least two days regardless of the state. The control group was participants who were on a regular diet or supplied with placebo.

### 2.4. Study Selection

Other articles related to the purpose of this study were retrieved by snowballing; the reference lists of included studies were also searched. To avoid duplicates or potential missing articles, two independent reviewers (M.L. and S.W.) evaluated studies against the inclusion criteria independently. All titles and abstracts were cross referenced to verify the rationality of each determinant and to identify studies for full-text review after eliminating duplicates. Inconsistencies were resolved by a third experienced researcher (H.F.) according to the inclusion/exclusion criteria. In this sense, the Cohen’s kappa coefficient (indicating interrater reliability) between authors was over 90.

Articles included had to meet the following inclusion and exclusion criteria: (1) original research paper; (2) participants were 60 years or older; (3) scientific evaluation of outcomes; (4) study design was a randomized controlled trial (RCT) or non-randomized studies of the effects of interventions (NRSI). Articles were excluded if they did not meet the inclusion criteria and met any of the following criteria: (1) study design was a case report, intervention study, opinion letter, review, systematic review, or meta-analysis; and (2) participants with enteral tube feeding or appetite-affecting disorder. Meanwhile, all studies that used ONS (including studies combined with dietary counseling and/or physical activity) were included in the current review regardless of the type or dosage, such as those containing micronutrients (fat, carbohydrate, protein, vitamins, and minerals). The PICO (population, interventions, comparators, outcomes) criteria statement for inclusion is presented in Table 1.

### 2.5. Outcome Measures

The primary outcome was appetite. This included overall appetite, hunger, fullness, desire to eat, “how much do you think you can eat now?”, and “how preoccupied are you with thoughts of food?”. Secondary outcomes were intake (including overall energy intake, protein intake, fat intake, and carbohydrate intake), body weight, body mass index (BMI), diarrhea, pressure sores, quality of life (QoL), and total health care cost indices. The measurement methods of the energy intake are reported in the Supplementary Materials (Table S2). Results were standardized when they appeared differently.

### 2.6. Data Extraction

A pre-determined data extraction table was used to capture all key characteristics and outcomes (including author, year of publication, country, study design, intervention length, settings, participants, participant situation, age, interventions, control and effect of the interventions). Meanwhile, the nature of ONS used in the included studies were also recorded in Table 2. Two researchers independently performed information extraction and the risk of bias assessment for included full-text articles; disagreements were solved with the help of a third experienced researcher.

For articles that reported outcomes at multiple time points, data were extracted at each time point [49,50], for trials with a crossover design [51], only the first phase data were used in the meta-analysis. Moreover, if a study combined ONS with other interventions [40,52], only individual outcomes of ONS were extracted. When no relevant value was reported, the results were included in the systematic review.

### 2.7. Statistical Analyses

Relevant data were extracted using RevMan 5.4 software (Copenhagen: The Nordic Cochrane Centre, The Cochrane Collaboration, 2014) [53]. The mean difference (MD), standard mean difference (SMD), and 95% confidence intervals (95% CIs) were used as numerical variable effect indicators. For dichotomous outcomes, we calculated the overall RR, while for continuous outcomes we calculated Hedges’ g. The between-study variation was estimated using a restricted maximum likelihood approach.

Heterogeneity was evaluated by Q test and I^2^ statistic: *p* > 0.1 and I^2^ ≤ 50% were judged as no heterogeneity, and the fixed model (FM) was used for the data analysis; *p* ≤ 0.1 and I^2^ > 50% were judged as the presence of heterogeneity, and the random model (RM) was used. When the data were over 10-fold different in mean or difference, we chose SMD as the effect size (SE) indicator. A known barrier for ONS used among the elderly is the volume that needs to be consumed, which may result in low compliance and reduction in effectiveness [54]. Therefore, subgroup analyses were conducted according to the nutrient density. We did not define the density but followed the definition of the included RCTs. Results of the meta-analysis were visualized using forest plots, while the publication bias was assessed using the Egger’s test.

### 2.8. Assessment of Risk of Bias and Study Quality

**Figure 1:**
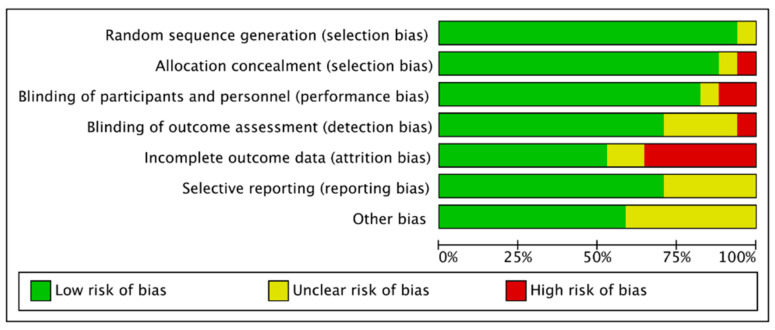
Risk of bias graph: judgements for risk of bias items presented as percentages across all included studies.

The methodological quality and risk of bias were assessed using the Cochrane Collaboration guideline by two independent reviewers (M.L. and X.Y.) [55]. Six domains were included: selection bias, performance bias, detection bias, attrition bias, reporting bias, and other types of bias. The bias was low when the question was answered “Yes”; the bias was high when it was “No”; while “Unclear” meant that the possible bias was connected to a lack of information or uncertainty. The full details of each study and domains are showed in Figure 1.

## 3. Result

### 3.1. Systematic Review

**Figure 2:**
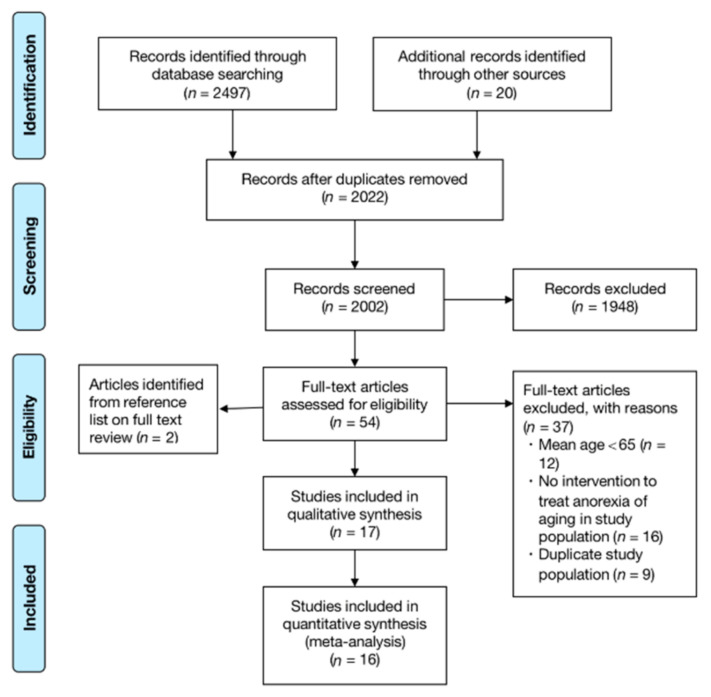
Flow diagram for screening and eligibility of studies for inclusion.

The flowchart of systematic review and meta-analysis is shown in Figure 2. A total of 2497 records were found through systematic search, and additional records were identified through other sources (search of gray databases, relevant systematic reviews, reference lists) (*n* = 20). After removing duplicates, 2022 records remained. By screening the titles and abstracts, 54 citations were full-text viewed and 37 articles were excluded (mean age < 65 (*n* = 12); there was no intervention in the treatment of AA (*n* = 16); duplication (*n* = 9)). More information is shown in Figure 2. For those in which outcome data were not available, we contacted the authors and partially obtained the corresponding responses. Eventually, 17 articles published from 1994 to 2016 were included in the systematic review and meta-analysis (*n* = 1204). One article reporting only the abstract was included for the data of energy intake after obtaining the detailed information of the full-text [56].

### 3.2. Study Characteristics

Characteristics and demographic information of the included RCTs are described in Table 2. Five studies were conducted in Sweden [51,57,58,59,60], four in France [40,49,50,52], three in the Netherlands [61,62,63], two in Germany [54,64], one in Australia [65], one in the United Kingdom [56], and one in Belgium [66]. There were no ethnicity differences mentioned in the study population. The mean age was 81.9 years (range: 74–87 years) and the mean BMI was 21.8 kg/m^2^ (range: 19.2–25.9 kg/m^2^); 72.5% of participants were female (range: 33–100%) with two gender-specific (female) trials included [58,65]. All included subjects had certain underlying disease or malnutrition. The intervention length ranged from two days to six months. Twelve studies intervened over one month [49,50,51,54,57,58,59,61,62,63,64,66]. The interventions were conducted generally in hospitals [40,52,60,65,66], communities [49,56,58,59,61,62] and nursing homes [50,51,54,57,63,64].

### 3.3. ONS Characteristics

Out of the 17 studies, one used solid ONS [50], four used both solid and liquid ONS [57,61,62,64], while the rest used liquid ONS. At the same time, one study used liquid ONS in combination with medication [58], one combined liquid ONS with nutritional education [59], and one with exercise [62]. 

The frequency of supplementation varied from one to three times a day, and the times were during breakfast [50], following breakfast [52], following breakfast (or lunch and dinner) [40], before lunch [65], after lunch [49], at the same time as the pharmaceutical prescriptions [51,59,60], between meals [54,63,64], in the evening [57], or any time [62]. The daily energy provided ranged from 200 to 600 kcal; details of the ONS characteristics, content, supplementation method, energy amount, executive personnel, and place are shown in the Supplementary Materials (Table S3). Two articles used high-volume ONS [60,62], while two used low-volume ONS [54,63], to distinguish the effect of energy on appetite, intake and weight, subgroup analyses were conducted when it comes to the different volume.

### 3.4. Methodological Quality of the Included Studies

**Figure 3:**
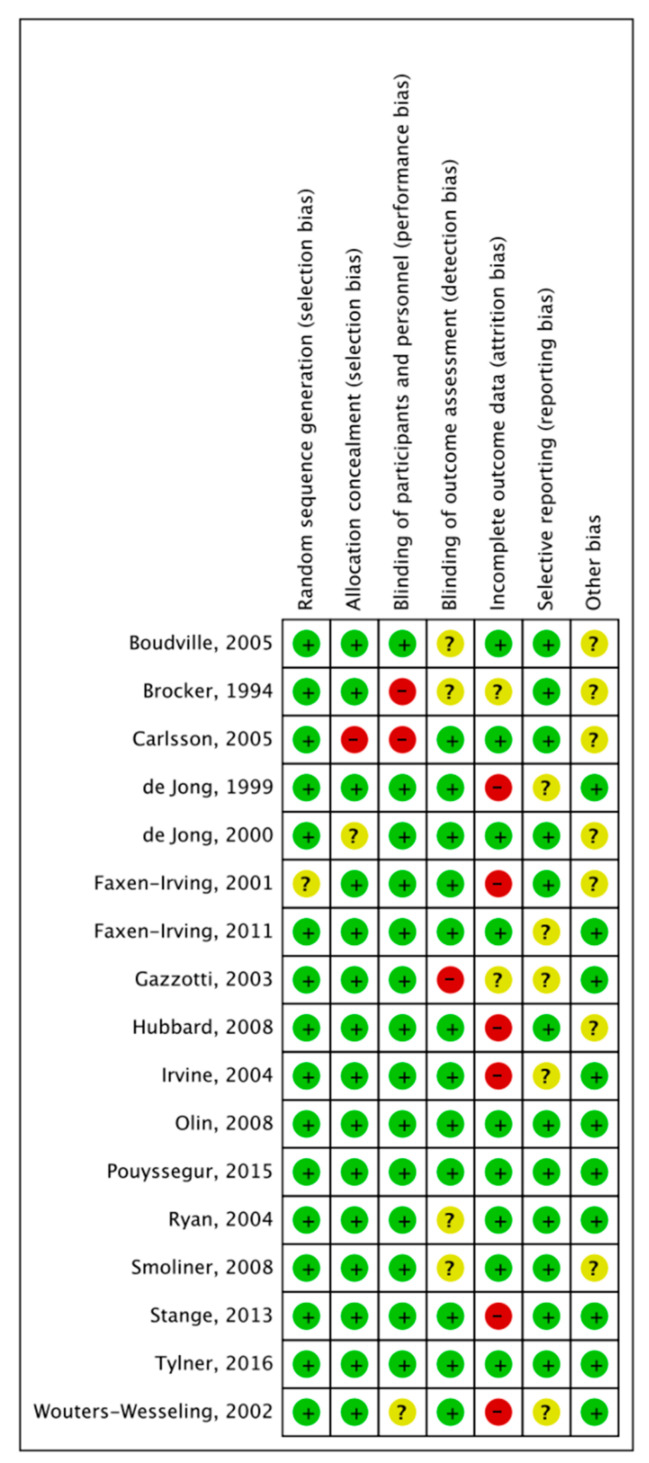
Risk of bias summary: judgements about each risk of bias item for each included study.

In terms of the methodological quality of the included studies, one study did not fulfill the criteria of random sequence generation and two did not fulfill the allocation concealment. Three of the studies did not blind participants or personnel. Additionally, five of the studies did not include the selective reporting. The details of domains are shown in Figure 3.

### 3.5. Effect of ONS on Appetite

Eight of the included studies reported a change of appetite [40,49,50,51,52,60,62,65]; visual analogue scales (VASs) were used in all studies to measure the subjective appetite [49,50,51,52,60,65], except for one which used a Likert scale (LS) with verbally labelled answering categories [62]. Overall appetite [49,50,60,62], hunger [51,60,62,65], fullness [51,60,65], desire to eat [51,60,65], “how much do you think you can eat now?” (consumption) [51,60], and “how preoccupied are you with thoughts of food?” (preoccupation) [51,60] were reported. The measurements of subjective appetite are summarized in the Supplementary Materials (Table S4).

For the different scores due to the different measurement scales, we performed a uniform unit conversion to unify the scores to the same scale (0–10). For studies that reported credible values, meta-analysis was conducted. The converted values included in the meta-analysis are shown in the Supplementary Materials (Table S5).

#### 3.5.1. Effect of ONS on Overall Appetite

**Figure 4:**
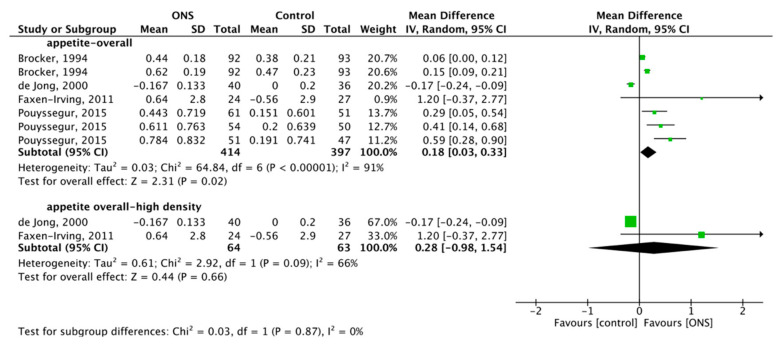
Forest plot comparing the effect of ONS on overall appetite and subgroup analysis.

Four articles reported the overall effect of ONS on appetite, with a total of 811 subjects included. The heterogeneity test showed substantial heterogeneity (*p* < 0.001, I^2^ = 91%), so the RM was used for meta-analysis, and the combined effect difference was significant (MD = 0.18, 95% CI (0.03, 0.33), *p* = 0.02), which showed that ONS had a positive effect on overall appetite (Figure 4). Subgroup analysis of two high-density groups with a total of 127 subjects studied [60,62]. RM was used due to the substantial heterogeneity (*p* = 0.09, I^2^ = 66%), and the combined effect difference was not significant, MD = 0.28, 95% CI (−0.98, 1.54), *p* = 0.66 (Figure 4), which showed that high-density ONS had no positive effect on appetite. 

#### 3.5.2. Effect of ONS on Hunger

**Figure 5:**
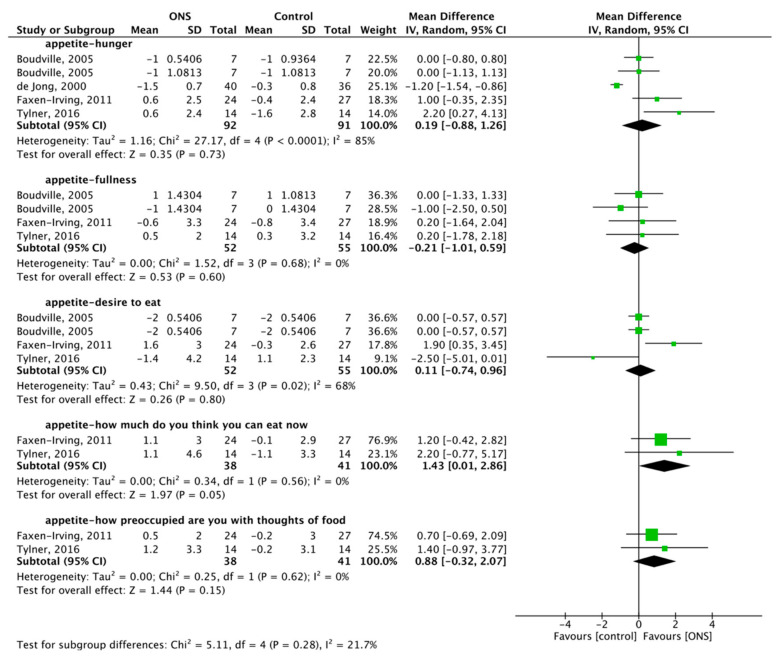
Forest plot comparing the effect of ONS on hunger, fullness, desire to eat, “how much do you think you can eat now?”, and “how preoccupied are you with thoughts of food?”.

Four articles reported the effect of ONS on hunger, with a total of 183 subjects included [51,60,62,65]. The RM was used for meta-analysis, and the ONS on hunger was 0.19, 95% CI (−0.88, 1.26), *p* = 0.73 with substantial heterogeneity (*p* < 0.001, I^2^ = 85%) (Figure 5). The results showed that ONS did not increase the feeling of hunger. 

#### 3.5.3. Effect of ONS on Fullness

Three articles reported the effect of ONS on fullness, with a total of 107 subjects included [51,60,65]. FM was used for meta-analysis, and the ONS on fullness was −0.21, 95% CI (−1.01, 0.59), *p* = 0.60 with no heterogeneity (*p* = 0.68, I^2^ = 0%) (Figure 5). The results showed no positive effect of ONS on fullness. 

#### 3.5.4. Effect of ONS on Desire to Eat

Three articles reported the effect of ONS on desire to eat, with a total of 107 subjects included [51,60,65]. The FM was used for meta-analysis, and the ONS on desire to eat was 0.11, 95% CI (−0.74, 0.96), *p* = 0.80, with substantial heterogeneity (*p* = 0.02, I^2^ = 68%) (Figure 5). The results showed no positive effect of ONS on the desire to eat.

#### 3.5.5. Effect of ONS on Consumption

Two articles reported the effect of ONS on “how much do you think you can eat now?”, with a total of 79 subjects included [51,60]. FM was used for meta-analysis, and the ONS on consumption was 1.43, 95% CI (0.01, 2.86), *p* = 0.05, with no heterogeneity (*p* = 0.56, I^2^ = 0%) (Figure 5). The results showed a positive effect of ONS on consumption. 

#### 3.5.6. Effect of ONS on Preoccupation

Two articles reported the effect of ONS on “how preoccupied are you with thoughts of food?”, with a total of 79 subjects included [51,60]. FM was used for meta-analysis, and the ONS on preoccupation was 0.88, 95% CI (−0.32, 2.07), *p* = 0.15, with no heterogeneity (*p* = 0.62, I^2^ = 0%) (Figure 5). The results showed that ONS increased the preoccupation with food. 

### 3.6. Effect of ONS on Intake

Twelve of the included studies reported the overall energy intake [40,51,52,54,56,57,58,60,61,62,65,66], eleven reported the protein intake [40,51,52,54,57,58,61,62,64,65,66], six measured the fat intake [40,52,57,61,62,65], and six reported the carbohydrate intake [40,52,57,61,62,65]. Where relevant, the recorded energy intake levels were converted to kilocalories (kcal) in order to homogenize results.

#### 3.6.1. Effect of ONS on Overall Energy Intake (OEI)

**Figure 6:**
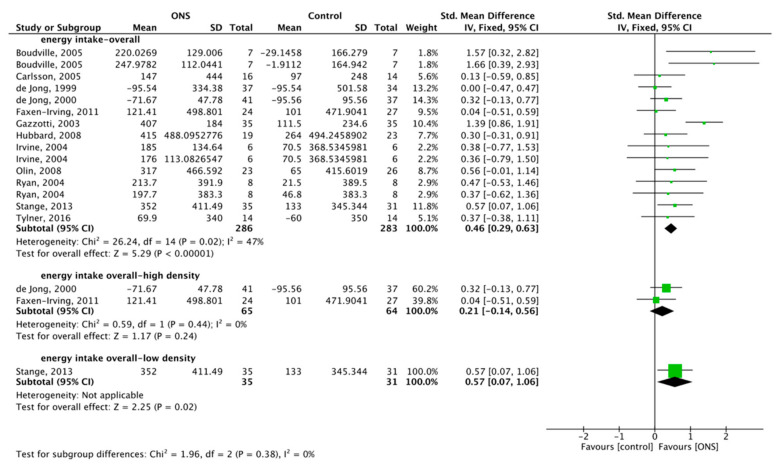
Forest plot comparing the effect of ONS on overall intake, and subgroup analysis.

A total of 569 subjects were included for meta-analysis. The heterogeneity test showed low heterogeneity (*p* = 0.02, I^2^ = 47%), FM was used, and the combined effect difference was significant, MD = 0.46, 95% CI (0.29, 0.63), *p* < 0.001, which showed that ONS had a positive effect on OEI (Figure 6). Subgroup analysis with a total of 129 subjects studied high-density ONS on OEI [60,62]. No heterogeneity (*p* = 0.44, I^2^ = 0%) was shown, so the FM was used, the combined effect difference was not significant (MD = 0.21, 95% CI (−0.14, 0.56), *p* = 0.24 (Figure 6)), and the results showed that high-density ONS had no positive effect on OEI. One low-density ONS affected OEI, with a total of 66 subjects studied [54]. By FM, the combined effect difference was significant (MD = 0.57, 95% CI (0.07, 1.06), *p* = 0.02 (Figure 6)), and the results showed that low-density ONS had no positive effect on OEI.

#### 3.6.2. Effect of ONS on Protein Intake

**Figure 7:**
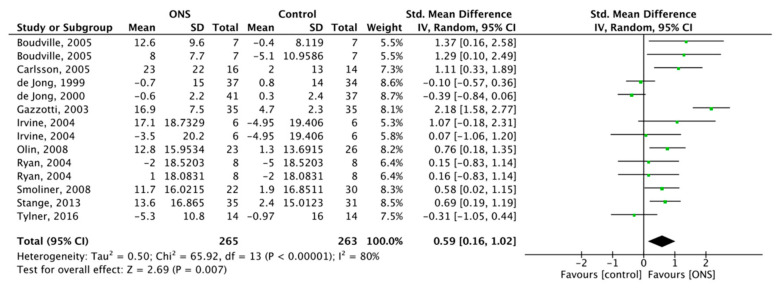
Forest plot comparing the effect of ONS on protein intake.

Eleven studies reported the effect of ONS on protein intake, with a total of 528 subjects included. RM was used for meta-analysis, and the ONS on protein intake was SMD = 0.59, 95% CI (0.16, 1.02), *p* < 0.007 with substantial heterogeneity (*p* < 0.001, I^2^ = 80%) (Figure 7). The results showed that ONS increased the intake amount of protein. 

#### 3.6.3. Effect of ONS on Fat Intake

**Figure 8:**
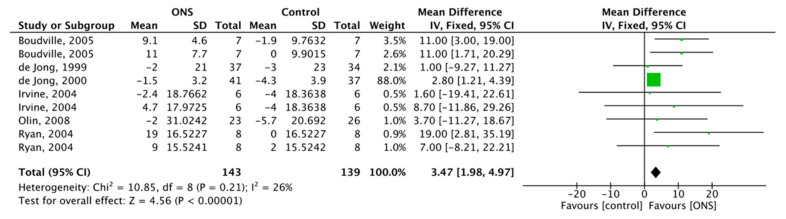
Forest plot comparing the effect of ONS on fat intake.

Six studies reported the effect of ONS on fat intake, with a total of 282 subjects included. FM was used for meta-analysis, and the ONS on fat intake was 3.47, 95% CI (1.98, 4.97), *p* < 0.001 with low heterogeneity (*p* = 0.21, I^2^ = 26%) (Figure 8). The results showed a positive effect of ONS on fat intake.

#### 3.6.4. Effect of ONS on Carbohydrate Intake

**Figure 9:**
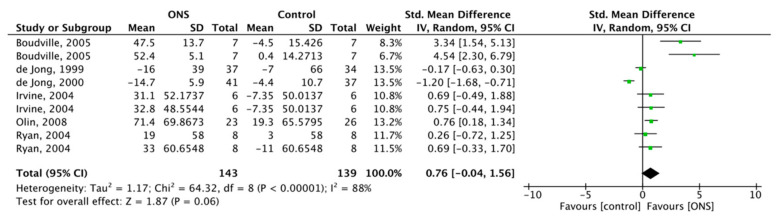
Forest plot comparing the effect of ONS on carbohydrate intake.

Six studies reported the effect of ONS on carbohydrate intake, with a total of 282 subjects included. FM was used for meta-analysis, and the ONS on carbohydrates intake was SMD = 0.76, 95% CI (−0.04, 1.56), *p* = 0.06 with substantial heterogeneity (*p* < 0.001, I^2^ = 88%) (Figure 9). The results showed no positive effect of ONS on carbohydrate intake. 

### 3.7. Effect of ONS on Body Weight

**Figure 10:**
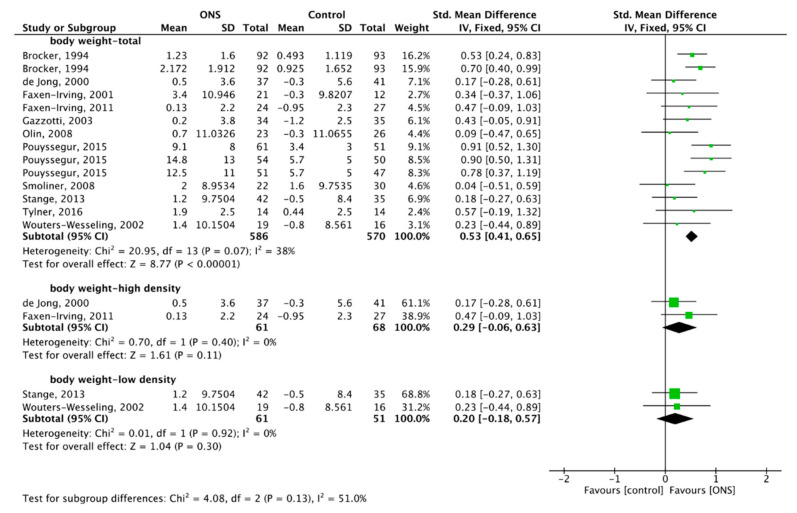
Forest plot comparing the effect of ONS on body weight, and subgroup analysis.

Eleven studies reported the effect of ONS on body weight [49,50,51,54,57,59,60,62,63,64,66]. A total of 1156 subjects were included in the meta-analysis. The heterogeneity test showed low heterogeneity (*p* = 0.07, I^2^ = 38%), so the FM was used for meta-analysis, and the combined effect difference was significant, SMD = 0.53, 95% CI (0.41, 0.65), *p* < 0.001, which showed that ONS increased the body weight of the elderly (Figure 10). 

Subgroup analysis was performed on the effect of two high-density ONS on body weight, with a total of 129 subjects studied. No heterogeneity (*p* = 0.40, I^2^ = 0%) was shown, so the FM was used, and the combined effect difference was not significant, SMD = 0.29, 95% CI (−0.06, 0.63), *p* = 0.11 (Figure 10), and the results showed that high-density ONS had no positive effect on body weight. Two low-density ONS on body weight were analyzed, with a total of 112 subjects studied. By FM, the combined effect difference was significant (SMD = 0.20, 95% CI (−0.18, 0.57), *p* = 0.30 (Figure 10)), and the results showed that low-density ONS had no positive effect on body weight.

### 3.8. Effect of ONS on BMI

**Figure 11:**
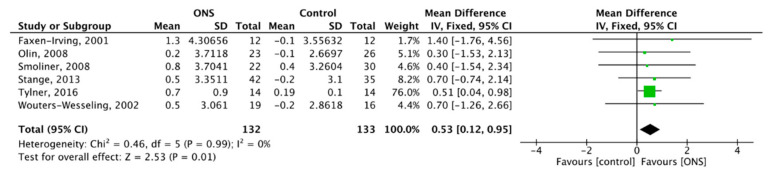
Forest plot comparing the effect of ONS on BMI.

Six studies showed the effect of ONS on BMI [51,54,57,59,63,64], with a total of 265 subjects included. FM was used for meta-analysis, and the ONS on BMI was MD = 0.53, 95% CI (0.12, 0.95), *p* = 0.01 with no heterogeneity (*p* = 0.99, I^2^ = 0%) (Figure 11). The results showed a positive effect of ONS on BMI.

### 3.9. Effect of Other Parts

Research showed that the use of ONS can relieve pressure ulcers in the elderly [50], and reduce the number of diarrhea events [50,63]. Surprisingly, solid cookie supplementation had a positive effect on reducing both diarrheal conditions and pressure sores (*p* = 0.027, 0.031, respectively) [50]. The ONS group also showed significant improvements in quality of life (QoL), and reduction in total health care cost indices by 37% [46,49,54,67,68]. 

To summarize all the results, the overall effect of ONS on the included outcomes has been reported (Table 3).

## 4. Discussion

This is the first systematic review and meta-analysis at this scale to critically synthesize the impacts of ONS on AA. We searched all the possible RCTs to fulfill the purpose of the review. The results suggested that AA is an important and indispensable issue, and the use of ONS can improve the three main aspects that affect the nutritional status, namely appetite, intake, and body weight. Meanwhile, the current study also found that ONS can increase BMI and QoL, and decrease pressure sores, diarrhea, and health care costs of AA. 

As the main feature of anorexia, appetite controls overall intake and therefore plays a pivotal role in the maintenance of nutrition [69], the results of the current meta-analysis showed the significance of ONS on overall appetite, but no positive effect on other parts of appetite assessment except for consumption. 

All the subjective appetite assessment methods used have been validated in previous studies [69,70,71]. Although appetite indicators can be considered similar with minor differences (see Supplementary Materials Tables S4 and S5), roughly score-based grade formats are difficult for determining values accurately, especially for older adults, and subtle differences in intake may largely affect nutritional status; therefore, future research should measure appetite more specifically. Even by using the same tools such as VASs, the scores can be categorized as low appetite (0–3), moderate appetite (4–6), and good appetite (7–10) [50], so appetite can be determined more precisely in statistical calculations [72,73].

Interestingly, the subgroup analysis of high- and low-density ONS of overall appetite showed that the energy intensity does not affect appetite. More updated evidence is needed to support the positive effect of ONS on appetite. In the meantime, although independent diet and improvement in nutritional status for AA can increase therapeutic effect and immunity [36,74], it is not the first choice to do these in an unacceptable way, such as parenteral nutrition [75]; at this point, the improvement of appetite is rather important [76]. Meanwhile, subgroup analysis regarding overall appetite, overall intake, and body weight found that subgroup analysis reduced heterogeneity, indicating that energy density was a cause of heterogeneity, but the results of subgroup analysis were not always positive, so the density of ONS was not very related to whether anorexia was improved or not.

Taking ONS before meals (30 min or 90 min), during pharmaceutical prescriptions, or between meals did not avoid a satiety effect on normal food intake [65]. The classical definition of satiety suggests that it leads to the termination of eating and is accompanied by appetite satisfaction, so the timing of supplementation is of great importance in AA [57]. The evening supplementation is not recommended because fluid ONS can lead to increased nocturnal urination in older adults [69].

Positive results of a three-day RCT suggested that high-carbohydrate and high-density ONS may reduce satiety and thus lead to an increase in intake [40], which was contrary to the results of the meta-analysis. In addition, subgroup meta-analysis of low-volume, nutrient- and energy-dense ONS also showed a slight positive effect on energy intake. These indicated that short-term but high-density ONS may lead to a transient increase in the intake of AA, but the long-term effects are not guaranteed [54]. With evidence suggesting that ONS should be taken for at least one month to show effectiveness, and the elderly had better compliance with higher energy-dense ONS [67], future designs could be more rigorous in terms of longer intervention duration and smaller dosages to improve the amount of intake and compliance of AA.

There was a synergistic effect with liquid/creamy dietary supplements [50]. The positive results of solid cookies suggested that appetite can be stimulated by vision, tactile, olfactory, and gustation [30,50]. Touching food through the fingers and chewing even in the absence of gingival issues can all stimulate the sense of touch; the sound of chewing may enhance the sense of hearing; and improving the sense of sight by beautifying the color and presentation of food are all valuable alternatives to achieve the physiological situation that refers to the body, the more one eats, and the more one wants to eat [50,77,78]. Studies contended that polydextrose can reduce the desire to eat, and therefore should be avoided especially in those with AA [46,69]. However, studies on AA are scarce, and well-designed RCTs are needed. The nutritional status of the elderly, even those showing malnutrition risk, should be strictly considered in future trials.

The flavor should be considered as the poor appetite of AA. ONS can be added to normal food, such as porridge or rice soup to improve the compliance of elderly who with moderate appetite. For those who are not satisfied with the taste, ONS can also be flavored with flavoring agents, such as cocoa or sesame powder [33,45,67]. Moreover, adding fruit as ONS are being brewed to make fruit-flavored shakes or yogurt may also increase the perception of food and increase the sense of consumption for AA [79,80]. The more severe and longer the malnutrition status was, the lower the initial energy should be given, usually starting at 10–15 kcal/(kg∙d), to prevent refeeding syndrome [30]. At the same time, special opportunities for eating should be placed on ONS to ensure more balanced and adequate nutrition when appetite is poor [80].

This study confirmed that ONS had a positive effect on the intake of protein and fat, as well as body weight and BMI, but was not positive on carbohydrate intake, perhaps because ONS contained major components of protein, amino acids, and fat, although they did not enhance appetite greatly; the autonomous intake and carbohydrate intake of the elderly was still low. Carbohydrates are the main component of the structure of living cells and are the main energy supplier, and have an important function in regulating cellular activity [81,82]. What is important for older people with anorexia is not to gain but to maintain weight, and this is more important for hospitalized patients [62]. Subsequent studies could research on improving carbohydrate intake of the elderly.

Although this paper showed positive results in multiple ways, older adults with AA have a lower appetite and daily intake, single nutritional supplement may not best enhance the nutritional status and the interest or enjoyment of food. Exercise, health education, fun activities, and psychological support can be combined with ONS to increase appetite and thus improve or even enhance their nutritional status [61].

## 5. Conclusions

In conclusion, the current research examined the effectiveness of ONS on AA and found that it can enhance appetite to some extent and has a positive effect on other major symptoms of AA, such as intake and body weight. Moreover, ONS reduced clinical outcomes such as pressure sores and diarrhea, as well as the healthcare costs. However, findings related to pressure sores and diarrhea should be interpreted with caution due to a lack of data; more studies are needed to investigate the impact of ONS in combination with other interventions on AA and the overall health of older adults.

## Figures and Tables

**Table 1:** PICO statement for studies’ inclusion.

Population	Older people (mean age over 60) in any settings, with any health conditions
Interventions	Treatment that used ONS of any kind
Comparators	Standard diets with or without placebo
Outcomes	With at least one assessment among appetite, intake, and weight

**Table 2:** Summary of the included articles.

Study (Author, Year, Country, Ref)	Study Design	Intervention Length	Setting	Participants	Participants Situation	Age ^#^	Interventions				Control	Effect of the Interventions
							ONS State	Times/Day	ONS Characteristic	Energy Amount		
Boudville, 2005, Australia [65]	Within-subject design	2–3 days (2 sessions)	Hospital	*n* = 14, F * = 100%, BMI = 22.6 (3.4)	Rehabilitation phase with an osteoporotic fracture	79 (7.5)	Liquid	Once	For elderly women	Contained 1046 kJ energy, 13 g protein, 11 g fat, 52 g carbohydrate in 250 mL by volume	Placebo	No change for appetite (*p* not reported)
Brocker, 1994, France [49]	RCT	4 months	Community (geriatric units)	*n* = 185, F * = U/K, BMI = 19.9 (2.6)	Recovering from acute illnesses	74 (8)	Liquid	Once	Ornithine oxoglutarate	/	Standard diet	Increase in appetite (*p* < 0.001) Increase in body weight (*p* < 0.01)
Carlsson, 2005, Sweden [58]	RCT	6 months	Not institutionalized	*n* = 45, F * = 100%, BMI = 20.4 (2)	Nondemented with a recent hip fracture	83 (5)	Liquid ONS/ in combination with medication	Once	/	200 ml/day corresponding to 836 kJ and 20g protein/day	Standard treatment	No change for appetite (*p* not reported) Increase in protein intake (*p =* 0.002)
De jong, 1999, the Netherlands [61]	RCT	17 weeks	Community	*n* = 145, F * = 70.3%, BMI = 24.3 (3.6)	Frail	78 (5.7)	Solid and liquid	/	/	The enriched products had an energy content of 0.48 MJ/product	Regular products and social program	No change for energy intake (*p* not reported)
De jong, 2000, the Netherlands [62]	RCT	17 weeks	Community	*n* = 159, F * = 71.1%, BMI = 24.5 (3.9)	Frail	78.7 (5.6)	Solid and liquid ONS + exercise	/	High-density ONS (rich in vitamins and minerals)	Two products per day delivered a mean energy intake of 0.48 MJ/d	Placebo	No change for appetite (*p* = 0.17) Increase in energy intake (*p* < 0.05)
Faxen-Irving, 2001, Sweden [59]	NRSI	6 months	Group-living, i.e., community assisted housing	*n* = 36, F * = 86%, BMI = 20.8 (3.2)	Demented	84 (4)	Liquid ONS and nutritional education	Twice	/	1720 kJ/day	Standard diet	Increase in body weight (*p* < 0.001) Increase in BMI (*p* < 0.001)
Faxen-Irving, 2011, Sweden [60]	RCT	8 days	Hospital	*n* = 51, F * = 53%, BMI = 21.3 (3.7)	Frail with several chronic disorders	84 (7)	Liquid	Three times	Energy dense oleic acid rich formula	A mix of 50% fat and 50% water that contains 466 kcal/100 mL from 60% monounsaturated fatty acids (MUFA), 10% saturated FA	Usual care	Increase in appetite (*p* = 0.021) Increase in energy intake (*p* < 0.0001) Increase in weight (*p* = 0.046)
Gazzotti, 2003, Belgium [66]	RCT	2 months	Hospital	*n* = 80, F * = 76%, BMI = 25.9 (5.1)	At risk of undernutrition	80.1 (6.9)	Liquid	Twice	/	200 mL sweet or salty sip feed twice daily of 500 kcal, 21 g protein per day	Standard diet	Increase in energy intake (*p* <0.01)
Hubbard, 2008, United Kingdom [56]	RCT	4 weeks	Community	*n* = 42, F * = U/K, BMI = 20.9 (3.5)	At medium or high risk of malnutrition	84 (7)	Liquid ONS and dietary advice	Three times	/	1674 kJ/day of the energy-dense supplement	Standardized diet	No change for appetite (*p* not reported) Increase in energy intake (*p* = 0.009)
Irvine, 2004, France [52]	Within-Subject design	3 days	Hospital	*n* = 12, F * = 33%, BMI = 21.3 (2.4)	Mildly undernourished people with disease	84 (7.8)	Liquid	Once	Protein-rich	HP: 250 kcal, 20 g protein, 1.5 g fat LP: 250 kcal, 3.5 g protein, 8.8 g fat	Usual care	Increase in energy intake (*p* < 0.01) Increase in appetite (*p* not reported)
Olin, 2008, Sweden [57]	NRSI	6 months	Nursing homes (frail elderly service flat)	*n* = 49, F * = 71%, BMI = 23.1 (2.2)	Need regular assistance	84 (6.2)	Liquid and solid	Once	Evening supplement	The evening meal contained an average of 530 kcal, 20 g protein	Regular meals	Increase in protein intake (*p* < 0.01) Increase in carbohydrates intake (*p* < 0.001) Increase in energy intake (*p* = 0.15) No change for weight (*p* = 0.72)
Pouyssegur, 2015, France [50]	RCT	6 weeks	Nursing homes	*n* = 154, F * = 80%, BMI = 19.2 (2.9)	Malnourished	86 (7.1)	Solid	/	Cookies	Total 52 g of cookies: 11.5 g of protein and 244 kcal daily	Standard institutional diet	Increase in weight (*p* = 0.038) Increase in appetite (*p* = 0.009)
Ryan, 2004, France [40]	Within-Subject design	3 days	Hospital	*n* = 16, F * = 38%, BMI = 20 (3)	Malnourished	77 (8)	Liquid	Once	2 different sets of volume of fat and carbohydrate	Contained 1050 kJ/250 mL (fat:carbohydrate:protein was 70:25:5 or 25:70:5)	Usual care	Increase in appetite (hunger) (*p* = 0.07) Increase in energy intake (*p* = 0.0035)
Smoliner, 2008, Germany [64]	RCT	12 weeks	Nursing homes	*n* = 65, F * = 71%, BMI = 21.6 (3.6)	Malnourished or at risk of malnutrition	85.2 (9.5)	Solid and liquid	/	/	The approximate nutrient content of the standard diet was 200 kcal of energy, 80g of protein, 60 g of fat, and 260 g of carbohydrates	Standard diet	Increase in protein intake (*p* = 0.007)
Stange, 2013, Germany [54]	RCT	12 weeks	Nursing homes	*n* = 77, F * = 91%, BMI = 21.5 (2.6)	Malnourished or at risk of malnutrition	87 (6)	Liquid	Twice	Low-volume, nutrient- and energy-dense	125 mL ONS of 600 kcal, 24 g protein per day	Routine care	Increase in energy intake (*p* < 0.001) Increase in protein intake (*p* < 0.001) Increase in weight and BMI
Tylner, 2016, Sweden [51]	RCT with crossover	12 weeks	Care residential homes	*n* = 39, F * = 60%, BMI = 23 (3.7)	Frail, malnourished or at risk of malnutrition	84 (7)	Liquid	Three times	ONS with oleic and linoleic acids, proteins, trace elements	Daily dose of 90 mL contributed with 360 kcal, 4.5 g protein	Usual care	Increase in energy intake Increase in weight Increase in appetite (*p* < 0.05)
Wouters-Wesseling, 2002, the Netherlands [63]	RCT	12 weeks	Nursing homes	*n* = 35, F * = 88%, BMI = 20.7 (2.9)	Demented, psycho-geriatric, at risk of undernutrition	82 (8.6)	Liquid	Twice	Low-volume	Complete micronutrient-enriched liquid nutrition supplement of 125 mL and 0.6 MJ	Placebo	Increase in weight (*p* = 0.02) Increase in BMI (*p* not reported)

BMI, body mass index (kg/ms^2^); NRSI, non-randomized studies of the effects of interventions; ONS, oral nutritional supplements; RCT, randomized controlled trial; U/K, unknown; * percentage of participants female; # reported as mean (standard deviation).

**Table 3:** The overall effect of the oral nutritional supplements (ONS) on the included outcomes.

**Primary Outcomes**	Appetite	Overall Appetite	Increased
		Hunger	No positive effect
		Fullness	No positive effect
		Desire to eat	No positive effect
		How much do you think you can eat now?	Increased
		How preoccupied are you with thoughts of food?	Increased
**Secondary Outcomes**	Intake	Overall energy intake	Increased
		Protein intake	Increased
		Fat intake	Increased
		Carbohydrate intake	No positive effect
	Body weight		Increased
	Body mass index (BMI)		Increased
	Diarrhea		Decreased
	Pressure sores		Decreased
	Quality of life (QoL)		Increased
	Total health care cost indices		Decreased

## Data Availability

Data are contained within the article.

## Supplementary Materials

The following are available online at https://www.mdpi.com/2072-6643/13/3/835/s1, Table S1. Database search formulas (example for 4 databases); Table S2. Measurement of food and energy intake; Table S3. Characteristics of the oral nutritional supplements (ONS) in the included studies; Table S4. Characteristics of the measurement of subjective appetite in the included studies; Table S5. Converted values included in the meta-analysis of appetite.
