# Supplementary material for: Effectiveness of Oral Nutritional Supplements on Older People with Anorexia: A Systematic Review and Meta-Analysis of Randomized Controlled Trials
Source: Nutrients. 2021 Mar 3;13(3):835. doi: 10.3390/nu13030835 (PMC8001033; doi:10.3390/nu13030835)
Supplement: Supplementary file 1 [file nutrients-13-00835-s001.pdf]

Supplementary Materials

Table S1. Database search formulas (example for 4 databases)

PubMed	Search terms for query	Results
#1	“aging” (MeSH Terms) OR “aging” (All Fields) OR “ageing” (All Fields) OR “aged” (MeSH Terms) OR “aged” (All Fields) OR “older” (All Fields) OR “older people” (All Fields) OR “elderly” (MeSH Terms) OR “elderly” (All Fields) OR “older adults” (All Fields)	5,916,110
#2	“appetite” (MeSH Terms) OR “appetite” (All Fields)	47,371
#3	“energy intake*” (All Fields) OR “food intake*” (All Fields) OR “food intake*” (All Fields)	301,025
#4	“oral nutritional supplement*” OR “oral nutritional intervention*” OR “oral supplement*” OR “enriched meal*” OR “nutritional intervention*” OR “ONS”	95,371
#5	“anorexia” OR “anorexia of ageing” OR “elderly with anorexia” OR “older people with anorexia” OR “senile anorexia”	7,283
#6	“Randomized Controlled Trial*” [Publication Type]) AND ("Randomized Controlled Trial*"[Title/Abstract] OR “randomization” [Title/Abstract]	152,454

#7	#1 AND #2 AND #3 AND #4 AND #5 AND #6	116
Embase		
#1	‘anorexia of aging’/exp	1388
#2	‘oral nutritional supplement*’/exp	10,017
#3	‘older people':ti,ab,it OR ‘older people'/exp OR ‘older adult*':ti,ab,it OR ‘older adult*'/exp OR elderly/exp OR elderly:ti,ab,it OR aged/exp OR aged:ti,ab,it	4,948,072
#4	‘randomized controlled trial*':ti,ab,it OR ‘randomization*':ti,ab,it OR ‘randomized controlled trial*'/exp	948,576
#5	#1 AND #3	1,060
#6	#5 AND ([aged]/lim OR [very elderly]/lim)	589
#7	#6 AND #2 AND #4	101
The Cochrane library		
#1	MeSH descriptor: [anorexia of aging] explode all trees	81
#2	MeSH descriptor: [oral nutritional supplement*] explode all trees	2467
#3	Cochrane trials	1,728,081
#4	#1 AND #2 AND #3	6
China National Knowledge Infrastructure (CNKI) [Chinese]		
#1	‘oral nutritional supplement*’ (All Fields)	19

#2	aging" (All Fields) OR "ageing" (All Fields) OR "aged" (All Fields) OR "older" (All Fields) OR "older people" (All Fields) OR "elderly" (All Fields)	1986
#3	"anorexia of ageing" OR "elderly with anorexia" OR "older people with anorexia"	28
#4	#1 AND #2 AND #3	0

Table S2. Measurement of food and energy intake

Study (author, year)	Measurement methods
Boudville, 2005	The amount of food stuffs consumed during each meal was determined and energy and macronutrient intakes were calculated using the Nuttab nutrient database (SERVE 3.95, SERVE Nutrition Management System, St Ives, New South Wales, Australia).
Brocker, 1994	Nutritional status was estimated on the basis of variables that were simple to measure in community practices.
Carlsson, 2005	A dietary assessment was done by asking questions about the minimum daily intake of certain food items, chosen as important contributors of energy or other nutrients. The dietitian made an estimate of a normal day’s food consumption. The nutritional value of the ONS was included in the calculation of the dietary intake at 6 months. Intakes were calculated by a standardized software program: Kost & Naringsdata AB, Dietist program, Stockholm, Sweden (The Swedish National Food Administration, 2000).
De jong, 1999	Imated dietary record was obtained by three trained dietitians at baseline (wk 0) and in the last week of intervention (wk 17). During a home visit before the intervention period, dietitians provided subjects with a clear explanation of the way to record and estimate portion sizes in household measures. During a second visit, they checked the diary and weighed the portion sizes of the most frequently consumed foods in household measures. Food consumption data were coded (with frequent cross-checking by all three dietitians), and energy and nutrients were calculated with the computerized Dutch Food Composition Table of 1997 and a supplement (1995) for folate and vitamin B-12 (Stichting Nederlands Voedingsstofenbestand 1995 and 1997).

De jong, 2000	At baseline and in the last week of intervention, a 3 d (two weekdays and one weekend day; non-consecutive) estimated dietary record was collected by three trained dietitians. Food consumption data were coded (with a frequent cross-checking by all three dietitians) after which energy and nutrients were calculated with the computerized Dutch food composition tables (Stichting Nederlands Voedingsstofenbestand, 1997). The energy and macronutrient content of the intervention food products were included in the food consumption data.
Faxen-Irving, 2001	/
Faxen-Irving, 2011	Total food and fluid intake was recorded at day 2-3 after admission, i.e., when the fat emulsion supplementation had started, and at day 8-9 or prior to discharge. The registration was performed by the staff by means of food records routinely used at the ward, i.e., the proportion of ingested food was estimated in quartiles in relation to the portion that was served. Fluid intake was estimated in ml.
Gazzotti, 2003	Consumption of each portion of supplement and regular meals were measured by direct observation and recorded as all, three quarters, half, one-quarter or none of the portion.
Hubbard, 2008	/
Irvine, 2004	The weight of each food was recorded before and after meals using a Tefal kitchen scale (max 3 kg, precision71 g). All items were weighed including snacks and drinks. Total energy intake was the sum of energy intakes for each meal plus snacks. Macronutrient and energy intakes were calculated from the weight of food consumed, the recipe used for cooking (available daily from the food production unit in hospital) and French food composition tables.

Olin, 2008	Two methods were used for registration of the food intake. The first was a 24-hour recall the residents were asked to recall the type and amount of all food and fluid consumed at each eating occasion the previous day and night. The 24-hour recall was used as a checklist for a one-day estimated dietary record, which was performed within a few days after the recall. The investigators registered the food and fluid intake at all eating occasions during one day. A telephone call was made to those residents who stayed up late in the evening to inquire about any additional intake.
Pouyssegur, 2015	To compare the calorie supplement actually consumed from w0 to w6 between the two groups, dietary intakes were measured during 5 days (d0, d3, d6, d40 and d42). Food and beverage consumption was scored as the percentage of each serving actually consumed (score 0, 1/3, 2/3 and 1). In one nursing home, calorie count was done by the dietician in charge of the menus.
Ryan, 2004	All food and drink items (excluding water) were covertly weighed before and after each meal using standard electronic kitchen scales (accurate to 71 g). The corresponding energy (kJ) and macronutrient intake (g) of the amounts consumed, with and without the additional intake from the supplements, was calculated over the whole day using the meal analysis program Bilnut 4.0 (Nutrisoft, France).
Smoliner, 2008	The nursing home staff was trained for dietary assessment, and food intake was recorded in standard portions. Single components of meals in addition to snacks and drinks were recorded. A research assistant visited the wards weekly to control recording of food intake. Energy and nutrient analyses were performed with EbisPro 6.0, which is based on the German food code.
Stange, 2013	Food intake was recorded once, for 3 consecutive days, including 1 weekend day and 2 week days, 6 to 8 weeks after study start in participants of 5 of the 6 nursing homes. Trained research staff weighed and documented the amount of all foods offered and

	leftovers after the residents' meals and in-between meals, including ONS consumed on these days. Energy and nutrient intake was calculated using a nutrient-analyzing software (EbisPro Version 6.0; J.Erhardt, Stuttgart, Germany) which is based on the German Food Code (BLS II, 3).
Tylner, 2016	To assess energy and nutrient intake (protein, calcium, and vitamin D) a 3-day food and fluid record was performed. The staff at each residential home was trained in how to register food and fluid intake. Any prescription or intake of nutritional supplements was also registered.
Wouters-Wesseling, 2002	Before the intervention, food intake was assessed for three consecutive weekdays by a combination of weighed and unweighed dietary records by a trained dietician. The main meal was recorded by weighing the food if patients were served and by estimating portion sizes from standard measures if patients served themselves. Leftovers of meals were weighed or the proportion of the leftover in relation to standard measures was estimated. The intake from other meals during the day was a record in terms of current household measures and standard portion sizes reported by nursing staff.

Table S3. Characteristics of the oral nutritional supplements (ONS) in the included studies

Study (author, year)	Supply time	ONS Content	Supply method	Executive personnel	Executive place
Boudville, 2005	30min/90min before lunch (standard, buffet-style meal)	• a commercially available supplement drink<br>• unrestricted access to a variety of foods (including tomato, ham, cheese, chicken, crackers, bread, butter, fruits, biscuits that were offered in separate portions and quantities (weighed before and after the meals to determine how much was consumed)	• a normal breakfast, then study subjects were required to drink a nutritional supplement drink before standard, buffet-style meal, drink within 10 min	• trained research dietitian<br>• investigators (A. B.)	social room in the rehabilitation ward
Brocker, 1994	at the end of the mid-day meal	• a commercially available production regular use in the hospital (Resource, Novartis Nutrition)<br>• unrestricted access to a variety of foods including tomato, ham, cheese, chicken, crackers, bread, butter, fruits, biscuits that	• presented in the form of two 5 g sachets to be dispersed in 200 ml of flavored solution, or with yoghurt, a milk-based dessert or stewed fruit	• investigators (A. B.)<br>• the research dietitian	own home

		were offered in separate portions and quantities were weighed before and after the meals to determine how much was consumed			
Carlsson, 2005	/	• protein rich oral liquid supplement	• the patients were randomized to treatment with a protein-rich liquid supplement alone or in combination with nan drolone decanoate injections	• research nurses	ward
de Jong, 1999	/	• fresh 100 g servings of fruit-based products (two types each of both fruit juice and compote) and 100 g servings of dairy products (vanilla custard, two types of fruit yogurt and 75 g of cheese curd with fruits) were provided weekly	• consume two products a day, one from a series of fruit products and one from a series of dairy products	• trained dietitians	/
de Jong, 2000	any time	• a fruit-based category and a dairy category<br>• all subjects were asked to consume one product daily out of each category (one	• all subjects were asked to consume one product daily out of each category (one dairy product and one fruit-based product per d)	• researchers	homes

		dairy product and one fruit-based product per d)	• Within the two categories several products were developed		
Faxen-Irving, 2001	when the prescribed drugs were distributed	• two 200 ml oral liquid supplements daily<br>• a juicy supplement was given in-between meals in the afternoon and a balanced supplement was given in the evening when the prescribed drugs where distributed	• the care giver attended a 12-h educational programme about nutrition and diet for the elderly	• dieticians<br>• physicians<br>• external care personnel	community assisted housing units
Faxen-Irving, 2011	at the same time as the pharmaceutical prescriptions, i.e., at 7.00, 14.00 and 20.00	• a dose of 30 ml fat emulsion (Calogen, strawberry flavored)	• fat emulsion was distributed at the same time as the pharmaceutical prescriptions and the intake of the fat emulsion was registered daily	• study nurses	ward

Gazzotti, 2003	/	• one Clinutren soup (1 kcal/ml) and one Clinutren 1.5 (1.5 kcal/ml) (Nestle´ Clinical Nutrition, Brussels, Belgium)	• prescription of ONS throughout hospitalization and convalescence.<br>• nurses and patients (once at home) kept a daily record throughout the trial of the supplements taken and of spontaneous intakes	• nurses	ward
Hubbard, 2008	/	• energy-dense supplement (Calogen, Nutricia)<br>• dietary advice in the form of a standardized dietary advice sheet	/	/	/
Irvine, 2004	follow standard breakfast	• each studied under 3 conditions, in which they were given in random order at breakfast, and on consecutive days	• on the 3 separate days, subjects consumed an identical breakfast at 08:00, contributing 200 kcal (10 g fat, 1 g protein and 25 g carbohydrates). Lunch (12:30) and dinner (18:30) consisted of a starter, main meal with vegetables	/	ward

			and meat, a dairy product and dessert<br>• snacks were offered at 15:00, and thereafter on request		
Olin, 2008	at evening	• ONS were served in addition to their regular meals	• the residents were informed that they should eat their meals as usual and that the offered meal in the evening was an extra meal	• care givers	service complex restaurant
Pouyssegur, 2015	during the breakfast and/or during the snack	• received eight cookies daily, ingredients were wheat, flour, fresh butter, milk protein (casein), sugar, vanilla aroma, baking powder and salt<br>• no palm oil or other vegetable oil<br>• patients who were prescribed dairy dietary supplements continue to take them	• eat during or between meals or snacks	• participant<br>• nursing staff	nursing homes
Ryan, 2004	immediately following a standard hospital	• oral sip supplements, matched for energy density and taste<br>• snacks were available on request	• each patient was studied over a 3 consecutive day run consisting of 2 treatment (supplement given immediately after breakfast) and 1	/	ward

	breakfast (or lunch and dinner)		control (no supplement given) day, with treatment order being randomized across subjects using a Latin square design		
Smoliner, 2008	between meals	• diet with protein- and energy-enriched soups and sauces and two additional snacks high in protein and energy	• the residents were assigned to a group receiving the standard food of the nursing home or a group with a protein- and energy-enriched diet and snacks.	• nursing home staff	ward
Stange, 2013	between meals	• 2 bottles of ONS with low volume (125 ml per bottle) and high nutrient and energy density (Fortimel Compact, Nutricia GmbH, Erlangen, Germany<br>• 2.4 kcal/ml, 12 g protein and 300 kcal per bottle/per day, supplementary to regular meals	• care personnel were instructed to encourage residents to consume the amount offered, and to support compliance by varying flavors, providing smaller portion sizes more frequently, or by adapting the time of offering	• care givers	ward

Tylner, 2016	at the same time as the medication, i.e., at 8:00 am, 12:00 noon, and 8:00 pm	• strawberry-flavored fat emulsion, Calogen Extra (Nutricia Advanced Medical Nutrition, Schiphol, The Netherlands)	• routine nutritional care while be served energy- and protein-fortified food prepared in each of the care residential homes	• the staff at each residential home	rooms
Wouters-Wesseling, 2002	during daytime between main meals	• two different flavours (orange/peach and blackberry) supplements by Numico Research BV<br>• the composition includes energy, protein, carbohydrates, fat, vitamins and so on	• patients received per day two tetrapacks of either the liquid nutrition supplement or a placebo product in a blinded manner in addition to their regular dietary intake for a study period of 3 months<br>• patients were helped and encouraged by the nursing staff to drink the supplement	• nursing staff	nursing homes

HP=High protein; LP=Low protein; ONS=oral nutritional supplements.

Table S4. Characteristics of the measurement of subjective appetite in the included studies

Study (author, year)	Tools	Points	Assess method	Assess time	Assess content	Additional content
Boudville, 2005	LS	five-point	The subjects completed.	On four occasions immediately before and 10 min after the pre-meal drinks and immediately before and after the meals.	to assess hunger, thirst, fullness, prospective consumption ("how much they could eat") and nausea.	/
Brocker, 1994	VAS	0-100	/	/	validated 100-point VAS (Comparisons between scores given by the various practitioners could not be made because each treated only 10 patients).	/
Carlsson, 2005	VAS	0-3	An experienced hospital dietitian (PC) made a 20–30 min long interview of the	on the second post-operative day and 6 months later at the outpatient department.	The patients rated their appetite: 0=good appetite, 1=neither good nor poor appetite, 2=poor appetite but eating, 3=poor appetite and not eating. The	Executive personal: an experienced hospital dietitian (PC).

			patients about their appetite and food habits (the week before the fracture) on the second post-operative day and 6 months later at the outpatient department.		patients described if their meals were self-made and, by using familiar household measures, the size and composition of their meals.	
de Jong, 2000	LS	/	/	/	After reading the question, subjects had to score on a point LS with verbally labelled answering categories. An example of a question is the following statement: In former days my appetite was: 1. much better than nowadays, 2. better than nowadays, 3. the same as nowadays, 4. worse than	A higher score corresponded to a more positive feeling about their taste and smell perception, a better appetite and more feelings of hunger. Initially variables were calculated: present taste perception

					nowadays, 5. much worse than nowadays.	(eight items, range 8±40), present smell perception (three items, range 3±15), appetite (six items, range 6±30), daily feelings of hunger (nine items, range 9±45), present smell perception compared with the past (three items, range 3±15).
Faxen-Irving, 2011	VAS	0-10	Performed with the support of the study nurses (3 nurses)	Before lunch at first day of inclusion and at day 8 after inclusion.	Five questions were given, considered to evaluate various aspects of appetite, i.e., hunger and desire to eat. The questions were: 1. How hungry do you feel? 2. How full do you feel? 3. How strong is your desire to eat?	The patients in the intervention group were asked to rate the acceptance of the product on a scale ranging from 0 to 7, higher values

						4. How much do you think you could eat now? 5. How preoccupied are you with thoughts of food?	indicating better acceptance.
Irvine, 2004	VAS	/	/		VAS was completed every half hour from before breakfast until lunch and hourly thereafter until diner. A total of 10.5 h was monitored. One investigator (PI) measured the lines with a ruler, to the nearest mm.	Questionnaires consisted of an A4 sheet of paper with seven 100 mm lines corresponding to seven questions considering hunger, fullness, desire to eat, pre-occupation with food, thirst, stress and cold. Weighted at the end of each line were two extreme states in relation to the question asked. The two answers anchoring each end were "not at all" and "extremely". Patients were instructed to mark a point on the line between the two	Desire to eat, a psychological rather than a physical sensation, referred to their earning for some food, differing from "preoccupation with food" which was termed as an inability to think of anything else but food. Fullness (used proxy measure of satiety) was evaluated by the

				extremities that best described their sensation.	question "How full do you feel" and described as the pleasant state of contentment felt after eating.
Pouyssegur, 2015	NS	0-10	Rated by the participant or by the nursing staff.	from w0 to w6	0 (absolutely no appetite) to 10 (extremely good appetite)	
Tylner, 2016	VAS	0-10	Were asked to rate their appetite.	All assessments and blood samples were performed and collected at start and end of each study period.	/	/

VAS: Visual rating scale; LS: Likert Scales; NS: Numerical Scale.

Table S5. Converted values included in the meta-analysis of appetite

Study (author, year)	ONS			Control			Time	Points
	mean	SD	n	mean	SD	n		
Brocker, 1994	0.44	0.18	92	0.38	0.21	93	30d	0-100
Brocker, 1994	0.62	0.19	92	0.47	0.23	93	60d	
de Jong, 2000	-0.167	0.133	40	0	0.2	36	/	0-10
Faxen-Irving, 2011	0.64	2.8	24	-0.56	2.9	27	/	0-10
Pouyssegur, 2015	0.443	0.719	61	0.151	0.601	51	w0 to w6	0-10
Pouyssegur, 2015	0.611	0.763	54	0.2	0.639	50	w0 to w10	0-10
Pouyssegur, 2015	0.784	0.832	51	0.191	0.741	47	w0 to w18	0-10
